# Emergence of Colistin Resistance Gene *mcr-8* and Its Variant in *Raoultella ornithinolytica*

**DOI:** 10.3389/fmicb.2019.00228

**Published:** 2019-02-15

**Authors:** Xiaoming Wang, Yao Wang, Ying Zhou, Zheng Wang, Yang Wang, Suxia Zhang, Zhangqi Shen

**Affiliations:** ^1^Beijing Advanced Innovation Center for Food Nutrition and Human Health, College of Veterinary Medicine, China Agricultural University, Beijing, China; ^2^Beijing Key Laboratory of Detection Technology for Animal-Derived Food Safety, Beijing Laboratory for Food Quality and Safety, China Agricultural University, Beijing, China

**Keywords:** colistin resistance, *mcr-8*, *mcr-8.4*, β-lactamase, *Raoultella ornithinolytica*

## Abstract

Recently, a novel mobile colistin resistance gene, *mcr-8*, was identified in *Klebsiella pneumoniae*. Here, we report the identification of *mcr-8* and its variant, *mcr-8.4*, in *Raoultella ornithinolytica* isolates which also belong to Enterobacteriaceae family. The *mcr-8* gene was located on transferrable plasmids with difference sizes. Notably, the transferability of *mcr-8*-carrying plasmids was enhanced once they entered into *Escherichia coli* hosts and multiple β-lactamase genes could co-transfer with *mcr-8*. These findings expand our knowledge of *mcr-8*-carrying bacterial species.

## Introduction

Colistin (polymyxin E), a polypeptide antibiotic, was originally isolated from the soil bacterium *Paenibacillus polymyxa* subsp. *colistin* (Poirel et al., 2017). Colistin is effective against most Gram-negative bacteria and was considered as one of the last-resort antibiotics for the treatment of human infections caused by multidrug resistant Gram-negative bacteria, especially, carbapenem-resistant Enterobacteriaceae (CRE) (Li et al., 2006). However, in 2016, the first plasmid mediated colistin resistance gene *mcr-1* was identified in *Escherichia coli*, *Klebsiella pneumoniae* and *Pseudomonas aeruginosa* (Liu et al., 2016). To date, the *mcr-1* gene has been detected in Enterobacteriaceae isolated from food, animals, human and environment in over 50 countries across five different continents (Hembach et al., 2017; Huang et al., 2017). Subsequently, plasmid-mediated colistin resistance genes *mcr-2*, *mcr-3*, *mcr-4*, *mcr-5*, *mcr-6*, and *mcr-7* have been identified in various bacterial species from humans and animals (Partridge et al., 2018). Recently, we reported the identification of *mcr-8* located on an InFII-type conjugative plasmid in *Klebsiella pneumoniae* isolated from chickens and pigs in China (Wang et al., 2018).

*Raoultella ornithinolytica* is closely related to *Klebsiella* and belongs to Enterobacteriaceae family (Beye et al., 2018; Hajjar et al., 2018). *R. ornithinolytica* is usually found in animals, soil, and botanical environment. This organism caused human infections, initially rare, are increasing according to several reports (Sun et al., 2015; Ponce-Alonso et al., 2016; Beye et al., 2018). So far, multi-drug resistance has been detected in *R. ornithinolytica* (Walckenaer et al., 2004; Castanheira et al., 2009; Khajuria et al., 2013), including *mcr-1* positive isolates (Luo et al., 2017). Here, we report the emergence of *mcr-8* in *R. ornithinolytica*.

## Materials and Methods

### Bacterial Isolation and Identification

A total of 300 cloaca samples were collected from chicken in commercial poultry farms of Shandong Province, China, in 2016. All the samples were screened on the CHROMAgar Orientation agar plate (bioMérieux, Lyon, France) containing 2 μg/ml colistin. The identification of bacterial species was performed using MALDI-TOF MS (BruKer Daltonik, Bremen, Germany), and then confirmed by 16S rDNA sequence analysis as described previously (Zhang et al., 2015; Luo et al., 2017). The presence of *mcr* (*mcr-1* to *mcr-8*) in *R. ornithinolytica* was determined by PCR amplification and followed by Sanger sequencing as described previously (Wang et al., 2018).

Before collection the study samples, we have drafted an application “Detection of plasmid mediated colistin resistance genes of Enterobacteriaceae in Shandong, China,” within which chicken are designed to be used as research object in this antimicrobial resistance study. Those experiments are guaranteed to conduct in accordance with the principles of the Beijing Municipality Review of Welfare and Ethics of Laboratory Animals, as well as rules and regulations from China Agricultural University’s committee on animal welfare and ethics. Finally, this application was approved by committee on Animal Welfare and Ethics in China Agricultural University.

### S1-PFGE and Southern Blotting

S1 nuclease-PFGE and Southern blotting were performed to locate the *mcr-8* gene in both donor and recipient strains as described previously (Zheng et al., 2017). Briefly, agarose gel plugs embedded strains were digested with S1 nuclease (TakaRa, Dalian, China), and Southern blotting was performed using the DIG-High Prime DNA Labeling and Detection Starter Kit II (Roche Diagnostics). The genomic DNA of the *Salmonella enterica* serovar Braenderup H9812 strain restricted with *Xba*I was used as the DNA marker. The *mcr-8* probe was the one, which we previously reported (Wang et al., 2018).

### Conjugation Assay

The horizontal transferability of *mcr-8* was examined using conjugation assay with *E. coli* J53 (azide-resistant) or *E. coli* EC600 (rifampicin-resistant) as the recipient strain. Considering colistin resistance spontaneous mutants might be confused with colistin transconjugants, the conjugation assay with *E. coli* J53 were performed twice, first was selected on LB agar plates containing 4 μg/ml colistin and 100 μg/ml azide, second was selected on 16 μg/ml amoxicillin and 100 μg/ml azide LB agar plates. In parallel, QDRO1 and QDRO2, and recipient strains J53 were plated on conjugation plates as control. Transconjugants were confirmed by PCR targeting the *mcr-8* and β-lactamase genes, *bla*_TEM-1B_ and *bla*_OXA-1_ in QDRO1 and QDRO2 transconjugants, respectively, as well as *Xba*I enzyme digested pulsed field gel electrophoresis (PFGE). For analysis of the transfer ability of *mcr-8* in the same genus, we further performed conjugation assay using the above identified QDRO1 and QDRO2 transconjugants (T-QDRO1 and T-QDRO2) as donor strains and *E. coli* EC600 as recipient strain. The transfer frequency was calculated as the number of transconjugants per recipient as previous reported (Zhao et al., 2017).

### Antimicrobial Susceptibility Test

The MICs of wild strains and transconjugants to antimicrobial agents (listed in Table 1) were determined by broth microdilution method, and the results were interpreted according to CLSI and European Committee on Antimicrobial Susceptibility Testing (EUCAST). The *E. coli* ATCC 25922 was used as a quality control strain.

**Table 1:** The minimum inhibitory concentrations of tested antimicrobial agents for the studied bacterial isolates.

Bacterial isolate^1^	MICs (μg/ml)^2^									
	CST	PB	AMC	AZT	CAZ	GEN	TET	FFC	CHL	CIP
*R. ornithinolytica* QDRO1	**16**	**8**	**128/64**	**2**	**64**	**>512**	**>256**	**>256**	**>256**	**128**
T-QDRO1	**16**	**4**	**32/16**	**2**	**16**	0.25	0.5	4	4	0.004
*R. ornithinolytica* QDRO2	**8**	**4**	**128/64**	**8**	**32**	**>512**	**>256**	**>256**	**>256**	**128**
T-QDRO2	**8**	**4**	**32/16**	**4**	**16**	0.25	0.5	4	4	0.004
*R. ornithinolytica* QDRO3	**4**	**4**	**128/64**	**2**	**64**	**>512**	**>256**	**>256**	**>256**	**16**
*R. ornithinolytica* QDRO4	**8**	**8**	**64/32**	**2**	**32**	**>512**	**>256**	**>256**	**>256**	**128**
*R. ornithinolytica* QDRO5	**16**	**8**	**128/64**	**4**	**32**	**>512**	**>256**	**>256**	**>256**	**64**
*R. ornithinolytica* QDRO6	**8**	**8**	**64/32**	**4**	**32**	**>512**	**>256**	**>256**	**>256**	**32**
*R. ornithinolytica* QDRO7	**8**	**8**	**64/32**	**4**	**16**	**>512**	**>256**	**>256**	**>256**	0.008
*R. ornithinolytica* QDRO8	**16**	**16**	**128/64**	**4**	**32**	**>512**	**>256**	**>256**	**>256**	**64**
*R. ornithinolytica* QDRO9	**32**	**16**	**128/64**	**4**	**32**	**>512**	**>256**	**>256**	**>256**	**64**
*R. ornithinolytica* QDRO10	**64**	**32**	**128/64**	**4**	**64**	**>512**	**>256**	**>256**	**>256**	**128**
*R. ornithinolytica* QDRO11	**4**	**4**	**64/32**	**2**	**32**	**>512**	**>256**	**>256**	**>256**	**64**
*R. ornithinolytica* QDRO12	**8**	**8**	**128/64**	**4**	**32**	**>512**	**>256**	**>256**	**>256**	**128**
*R. planticola* QDRP1	**8**	**8**	**64/32**	**4**	**8**	**>512**	**>256**	**>256**	**>256**	**16**
*R. planticola* QDRP2	**4**	**4**	**64/32**	**4**	**16**	**>512**	**>256**	**>256**	**>256**	**8**
*R. terrigena* QDRT1	**2**	**2**	**32/16**	**4**	**8**	**>512**	**>256**	**>256**	**>256**	0.016


*^1^T-QDRO1 and T-QDRO2 represent the transconjugations of R.ornithinolytica QDRO1 and R.ornithinolytica QDRO2. ^2^Antimicrobial agents are abbreviated as follows: CST, colistin; PB, polymyxin B; AMC, amoxicillin-clavulanate; AZT, aztreonam; CAZ, ceftazidime; GEN, gentamycin; TET, tetracycline; FFC, florfenicol; CHL, chloramphenicol; CIP, ciprofloxacin. The bold numbers mean the isolates are resistant to the tested antimicrobial agent.*

### Genome Sequencing and Analysis of Antibiotic Resistance Genes

Genomic DNA of the isolates were extracted using the Wizard Genomic DNA Purification kit (Promega), then subjected to WGS on the Illumina HiSeq 2500 platform according to the manufacturer’s protocols, which produced 150-bp paired-end reads. For each isolate analyzed by WGS, at least 100-fold coverage of raw reads was collected. The draft genomes were assembled using CLC Genomics Workbench 9.0 (CLC Bio, Aarhus, Denmark). Reference sequences of antibiotic resistance genes were from database ARG-ANNOT (de Man and Limbago, 2016).

## Results and Discussion

### Presence and Location of *mcr-8* in *Raoultella spp*

**FIGURE 1:**
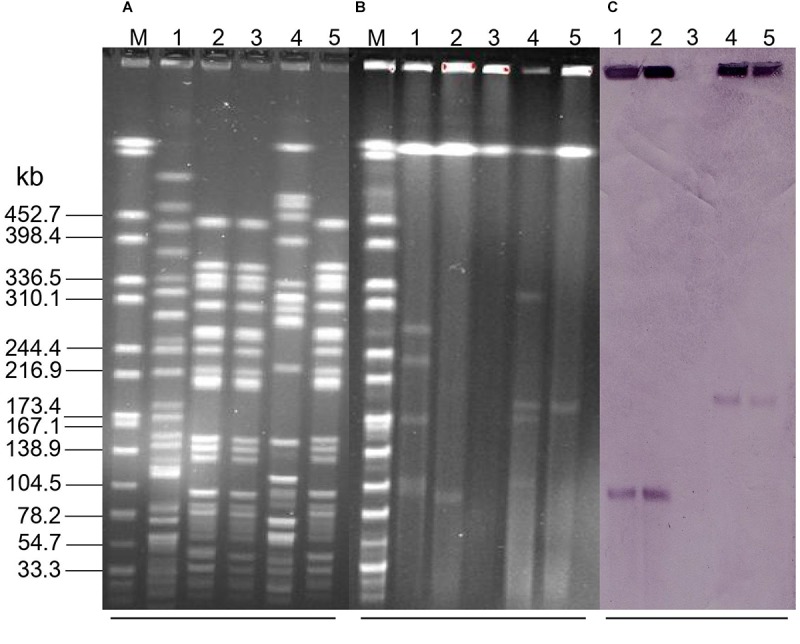
The location of *mcr-8* in *Raoultella ornithinolytica* QDRO1 and QDRO2 isolates and their transconjugants. **(A)** *Xba*I-digested PFGE of the *R. ornithinolytica* QDRO1 and QDRO2 isolates, transconjugants, and recipient *Escherichia coli* J53. **(B)** S1-PFGE and **(C)** the corresponding Southern hybridization using the *mcr-8*-specific probe. Lane M, marker H9812; Lane 1, *R. ornithinolytica* QDRO1; Lane 2, transconjugant T-QDRO1; Lane 3, recipient *E. coli* J53; Lane 4, *R. ornithinolytica* QDRO2; Lane 5, transconjugant T-QDRO2.

A total of 15 *Raoultella spp* strains obtained from 300 chicken cloaca samples, among which 12 *R. ornithinolytica*, 2 *R. planticola*, and 1 *R. terrigena*. PCR assays showed that two *R. ornithinolytica* strains, named QDRO1 and QDRO2, were positive for *mcr-8*, but no other *mcr* genes were identified in this 15 *Raoultella spp* strains. S1-PFGE and Southern blotting assay indicated that *mcr-8* were located on ∼90-kb and ∼200-kb plasmids in QDRO1 and QDRO2, respectively (Figure 1). These two *mcr-8*-carrying plasmids were named as pQDRO1 and pQDRO2, respectively.

### Transferability of *mcr-8* Gene

Conjugation assays showed that the pQDRO1 and pQDRO2 plasmids were transferable from *R. ornithinolytica* to recipient *E. coli* strains. The transfer frequencies of the pQDRO1 and pQDRO2 plasmids to *E. coli* J53 were 2.28 ± 1.64 × 10^-8^ and 1.71 ± 1.01 × 10^-8^, respectively. Meanwhile, transconjugants from amoxicillin and azide plates were resistant to colistin and *mcr-8* positive. These suggested that *mcr-8* was co-transferred with β-lactamase genes. As expected, donor strains QDRO1 and QDRO2, and recipient J53 did not grow on colistin and azide plates, or amoxicillin and azide plates. To determine whether the adaptation of *mcr-8-*carrying plasmids in *E. coli* could affect their transfer frequencies, we further performed the conjugation assays using the transconjugants as donor strains and *E. coli* EC600 as recipient strain. We found that the transfer frequencies of the pQDRO1 and pQDRO2 plasmids increased 10^3^ and 10^4^ folds, respectively, compared with the transfer frequencies of plasmids from *R. ornithinolytica* QDRO1 and QDRO2 to *E. coli*, respectively. To determine if the transfer frequencies of plasmids could be affected by the recipient bacteria, we performed the conjugation assays using the parental strains *R. ornithinolytica* QDRO1 and QDRO2 as donors and *E. coli* EC600 as recipients. The transfer frequencies of the pQDRO1 and pQDRO2 plasmids from *R. ornithinolytica* to *E. coli* EC600 were 4.17 ± 1.35 × 10^-7^ and 3.09 ± 1.29 × 10^-7^, respectively. We further performed the conjugation assays using the obtained transconjugants as donor strains and *E. coli* J53 as recipient strain. The transfer frequencies of pQDRO1 and pQDRO2 were 2.74 ± 1.31 × 10^-4^ and 3.71 ± 1.98 × 10^-4^, respectively. Similar to the previous results, increased transfer frequencies were observed for the pQDRO1 and pQDRO2 plasmids once they adapted to the *E. coli* host. These findings demonstrated that *mcr-8* gene is able to transfer between different bacterial species, which may further promote the dissemination of drug resistance.

### Antimicrobial Susceptibility

Antimicrobial susceptibility test showed that this 15 *Raoultella spp* strains were all resistant to colistin, polymyxin B, amoxicillin-clavulanate, aztreonam, ceftazidime, tetracycline, florfenicol, chloramphenicol, and only *R. ornithinolytica* QDRO7 and *R. terrigena* QDRT1 were sensitivity to ciprofloxacin (Table 1). Both transconjugants were not only resistant to colistin and polymyxin B, but also resistant to β-lactam antibiotics, such as amoxicillin-clavulanate, aztreonam and ceftazidime, which implied that β-lactamase producing genes might be co-transferred with *mcr-8*.

### Whole Genome Sequencing Analysis

WGS analysis showed that a 16.5-kb contig (GenBank: QWIX00000000) of *R. ornithinolytica* QDRO1 carrying *mcr-8* showed 100% query coverage and 99% identity to the corresponding segment of the *mcr-8*-carrying plasmid pKP91 from *K. pneumoniae* (Genbank number: MG736312) by Blastin in the NCBI database. A *mcr-8* variant, termed *mcr-8.4* (Genbank number: MH791448), was found in this 16.5-kb contig. Compared with *mcr-8*, *mcr-8.4* gene carried an A1209C transversion, which resulted in Serine to Arginine substitution. Similarly, the 25.5-kb *mcr-8-*carrying contig (Genbank number: MK097469) of *R. ornithinolytica* QDRO2 showed 83% query coverage and 99% identity to the corresponding segment of the *mcr-8*-carrying plasmid pKP91 from *K. pneumoniae*. Genetic structure analysis of the two *mcr-8*-carrying contigs showed that two copies of ΔIS*903B* located upstream and downstream of *mcr-8.4* in *R. ornithinolytica* QDRO1, while, only one copy of ΔIS*903B* located upstream of *mcr-8* in *R. ornithinolytica* QDRO2 (Supplementary Figure S1). Plasmid replicon type was carried out using the Center for Genomic Epidemiology^1^, and showed that *R. ornithinolytica* QDRO1 contained IncHI2, IncA/C2, IncX3, and IncFII-type plasmids, and *R. ornithinolytica* QDRO2 contained IncHI2, IncFIB, IncHI1B, and IncFII-type plasmids. To further identify the replicon type of plasmids pQDRO1 and pQDRO2, we detected the replicon genes, which found in wild strains, in transconjugants of *R. ornithinolytica* QDRO1 and QDRO2 using primers listed in Supplementary Table S1. Results showed that the plasmids pQDRO1 and pQDRO2 both belong to IncFII-type, which is same with plasmid pKP91.

Analysis of the whole genome sequences of QDRO1 and QDRO2 isolates showed that these two strains contained multiple resistance genes (Table 1). As shown, except *mcr-8.4*, *R. ornithinolytica* QDRO1 also contained *aadA1*, *aph(3′)-Ia*, *strA*, *strB*, *aac(6′)-Ib*, and *armA*, *fosA*, *mph(E)*, *floR*, *cml*, *qnrB4*, *sul*, *tet(B)*, *tet(34)*, *bla*_TEM-1B_, *bla*_OXA-1_, *bla*_DHA-1_. Similarly, except *mcr-8*, *R. ornithinolytica* QDRO2 contained *aac(3)-IVa*, *aph(4)-Ia*, *aadA2*, *fosA, mph*(A), *mph*(E), *cat*, *floR*, *cml*, *QnrS4*, *oqxAB, QnrB52*, *sul1*, *sul2* and *sul3, tet*(A), *tet*(34), *tet*(O), *tet*(B), *bla*_TEM-1B_, *bla*_OXA-1_, *bla*_SHV -73_.

Our above antimicrobial susceptibility assay suggests that β-lactamase genes might be co-transferred with *mcr-8*. In order to determine the co-transfer of these genes, PCR amplification was performed to detect the presence of β-lactamase genes in transconjugants using primers listed in Supplementary Table S1. *bla*_TEM-1B_ and *bla*_DHA-1_ were detected in QDRO1 transconjugant, while *bla*_OXA-1_ and *bla*_SHV -73_ were present in QDRO2 transconjugant. These findings indicated that *bla*_TEM-1B_, *bla*_DHA-1_, *bla*_OXA-1_, and *bla*_SHV -73_ could co-transfer with *mcr-8*.

## Conclusion

This study identified colistin resistance genes *mcr-8* and its variant, *mcr-8.4*, in *R. ornithinolytica*. The two *mcr-8*-carrying IncFII-type plasmids could be transferred to *E. coli* by conjugation. In addition, the transferability of the two plasmids were enhanced once they entered into *E. coli* hosts, which might further accelerate the dissemination of *mcr-8* among *Enterobacteriaceae*. It is worth noting that the co-transferability of *mcr-8* with several β-lactamase genes may further facilitate the dissemination of *mcr-8* among Enterobacteriaceae.

## Author Contributions

ZS, SZ, and YaoW conceived and designed the experiments. XW, YaoW, YZ, and ZW performed the experiments. ZS and XW analyzed the data. XW, YanW, and ZS wrote the manuscript.

## Conflict of Interest Statement

The authors declare that the research was conducted in the absence of any commercial or financial relationships that could be construed as a potential conflict of interest.

## Footnotes

1 http://genomicepidemiology.org/
