# Supplementary material for: Emergence of Colistin Resistance Gene mcr-8 and Its Variant in Raoultella ornithinolytica
Source: Front Microbiol. 2019 Feb 15;10:228. doi: 10.3389/fmicb.2019.00228 (PMC6384272; doi:10.3389/fmicb.2019.00228)
Supplement: Supplementary file 1 [file Data_Sheet_1.doc]

**Supplementary materials-Table S1**

Table S1 PCR primers and contidions used in this study.

| Primers | Sequence(5'-3') | Annealing  Temperature(℃) | Product  Size(bp) | Reference |
| --- | --- | --- | --- | --- |
| *bla*_DHA-1_-F | GCTGGGGTTATCTCACACCT | 55 | 410 | This study |
| *bla*_DHA-1_-R | TTGCGCCCGTTTTATGCAC |
| *bla*_TEM-1B_-F | CAACATTTTCGTGTCGCCCTT | 55 | 496 | This study |
| *bla*_TEM-1B_-R | TTCATTCAGCTCCGGTTCCCA |
| *bla*_OXA-1_-F | TTTCAAGATCGCATTATCACT | 50 | 563 | This study |
| *bla*_OXA-1_-R | CTTGATGTTAAATTCGACCC |
| *bla*_SHV-73_-F | CGCTTTCCCATGATGAGCACCT | 55 | 320 | This study |
| *bla*_SHV-73_-R | CGCCTCATTCAGTTCCGTTTCCC |
| IncHI2-F | CTGTTCGTCCCATCACCAAA | 55 | 484 | This study |
| IncHI2-R | GCTGCATCAAAACCTTATGATCG |
| IncA/C2-F | CGTAATCAAAGACTCACCGCAAA | 55 | 465 | This study |
| IncA/C2-R | CCGTCATCACTGATACATTCGAG |
| IncX3^a^-F | AACCTTAGAGGCTATTTAAGTTGCTGAT | 55 | 376 | 1 |
| IncX3-R | TGAGAGTCAATTTTTATCTCATGTTTTAGC |
| IncFⅡ^b^-F | CTGTCGTAAGCTGATGGC | 55 | 270 | 1 |
| IncFⅡ-R | CTCTGCCACAAACTTCAGC |
| IncFIB-F | GGAGTTCTGACACACGATTTTCTG | 55 | 702 | 1 |
| IncFIB-R | CTCCCGTCGCTTCAGGGCATT |
| IncHI1B^c^-F | GGAGCGATGGATTACTTCAGTAC | 55 | 471 | 1 |
| IncHI1B-R | TGCCGTTTCACCTCGTGAGTA |

1. Carattoli, A., Bertini, A., Villa, L., Falbo, V., Hopkins, K., Threlfall, E. (2005) Identification of plasmids by PCR-based replicon typing. *J Microbiol Methods* 63, 219-28. a: represents X primer in reference 1; b: represents FII_S_ primer in reference 1; c: represents HI1 primer in reference 1.


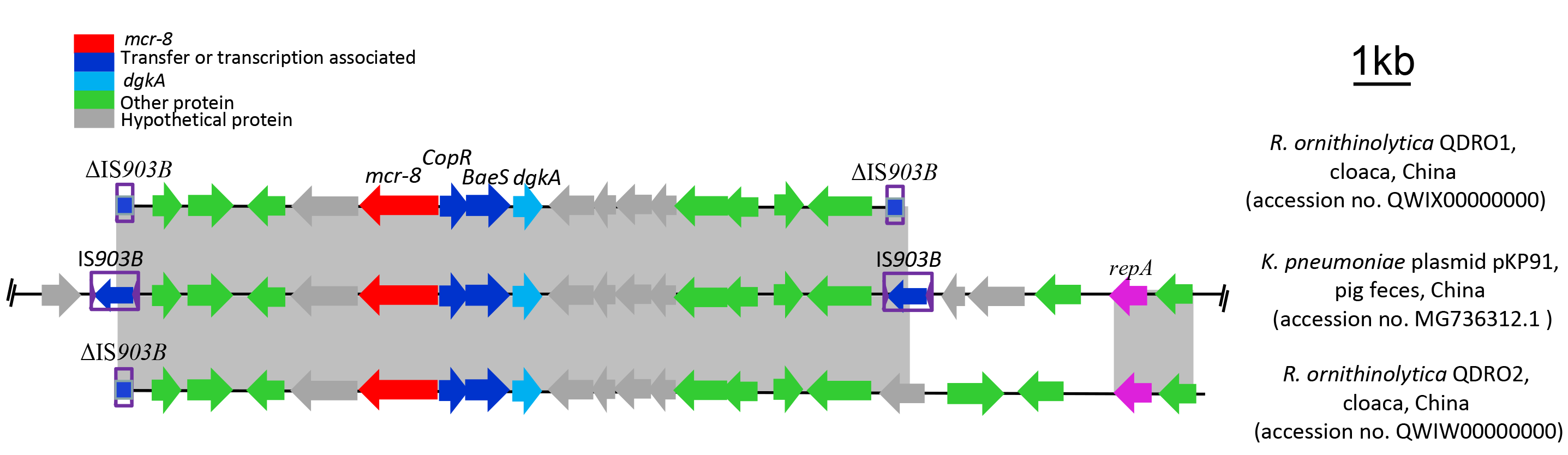


**Figure S1**. Genetic organization of scaffolds containing *mcr-8.4* and *mcr-8* obtained from *R. ornithinolytica* QDRO1 and QDRO1 in this study, and structural comparison with *K. pneumoniae* KP91 (GenBank accession no. MG736312.1). The positions and orientations of the genes are indicated by arrows, with the direction of transcription shown by the arrowhead. Gray shading indicates >99% nucleotide sequence identity.
